# Wooded biocorridors substantially improve soil properties in low-altitude rural benchlands

**DOI:** 10.1016/j.heliyon.2024.e24381

**Published:** 2024-01-17

**Authors:** Aleš Kučera, Dušan Vavříček, Daniel Volařík, Pavel Samec, Luboš Úradníček

**Affiliations:** a Department of Geology and Soil Science, Faculty of Forestry and Wood Technology, Mendel University in Brno, Zemědělská 3, 613 00, Brno, Czech Republic; b Department of Forest Botany, Dendrology and Geobiocoenology, Faculty of Forestry and Wood Technology, Mendel University in Brno, Zemědělská 3, 613 00, Brno, Czech Republic

**Keywords:** Agricultural landscapes, Carbon stock, Forest vegetation, Soil hydrolimit, Soil respiration

## Abstract

This study examines soil properties in 30- and 60-year-old agricultural biocorridors and provides a comparative overview with neighbouring farmland. Both mixed and undisturbed soil samples were collected from six farmland/biocorridor study areas to assess a wide spectrum of physical, hydrophysical, chemical and biological soil properties. Biocorridor soils were characterised by higher water retention capacities, porosity, aeration and soil carbon stock, the latter increasing with depth. On the other hand, biocorridor bulk density under forest vegetation cover was lower, indicating progressive soil restoration. Slightly lower soil reactions in biocorridor soils disproved the hypothesis that nutrient-rich soils under biocorridors would form substrates with a high base cation content, leading to soil acidification. Biological activity, expressed through respiration coefficients, was generally low due to unfavourable physical conditions (clayey or silty-clay substrates), with the lowest levels in biocorridors. Nevertheless, biocorridor soil microbiota displayed more effective utilisation of organic matter as a carbon and nitrogen source, with lighter-textured soils tending to show more effective organic matter utilisation after excluding the influence of land use. Our results confirm biocorridors as an important landscape component, contributing to both soil stability and local revitalisation of soil environments and further emphasising their potential as climate-change mitigation tools in their role as carbon sinks.

## Highlights

• Biocorridor soils have higher water retention, porosity, aeration and total organic carbon content.
• Biocorridor soils under forest vegetation have lower bulk density, indicating soil restoration.
• Soil microbiota in biocorridor soils utilise soil carbon and nitrogen more effectively.

## Graphical abstract

**Figure:**
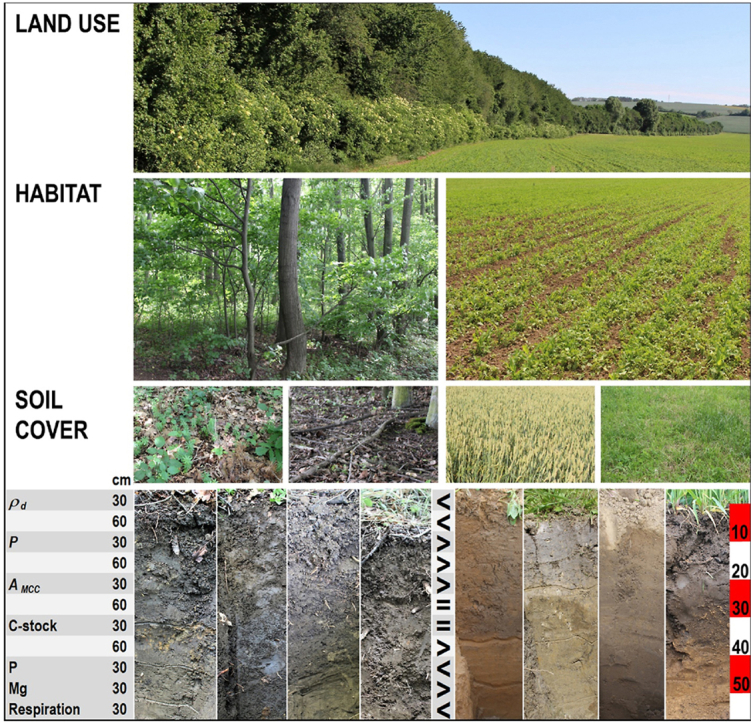

## 1 Introduction

Long-term application of conventional farming methods on agricultural land can result in degradation processes causing physical, chemical and biological changes to a considerable depth in the soil profile. Detailed global statistics of soil degradation sources are difficult to obtain due to i) a lack of precise definitions for describing the many factors affecting soil degradation, and ii) an insufficient understanding of the impacts of regional reclamation measures around the world. However, on a European level, over 1.114 million km^2^ of arable land is presently subject to differing levels of soil degradation [1].

Soil degradation processes may be affected by local mineralogy (e.g. fine-earth, stoniness), organic matter content, soil depth, soil morphostratigraphy, moisture regime and vegetation cover, particularly as regards forest floor production and its positive impact on erosion processes [[2], [3], [4], [5], [6]]. In addition, degradation may be enhanced by external factors, including anthropogenic impacts such as overuse of chemical fertilisers and pesticides [3] and the overuse of heavy machinery [7].

There has been a huge increase in the use of agricultural chemicals over the past 40 years [8]. Especially overuse of nitrogen-based fertilisers replaced more environmentally-friendly approaches based on application of organic matter, appropriate crop rotations and catch crops [9], even when such methods are known to provide improved soil structure, stable nutrient availability and better water retention capacity throughout the rooting zone [10]. Soil compaction by heavy machinery presently affects ca. 33 million ha of soil in Europe [2]. Compaction increases the proportion of capillary pores and soil bulk density, fundamentally altering its structure [11], water-air regime [12,13], infiltration capacity and hydraulic conductivity. These changes in turn negatively affect nutrient availability [14] and plant rooting [4,15], as well as soil biological activity and biodiversity [16]. Elimination of such degradation factors [17], along with the construction of remediation measures such as windbreaks, hedgerows and biocorridors, has been shown to promote soil regeneration, maintenance of soil functioning and ecosystem services and the soil's adaptation capacity.

In the Czech Republic, artificial biocorridors, classed as structural elements within the Czech ‘Territorial Systems of Ecological Stability’, have been planted since the 1970s, in part to create ecological networks allowing wildlife migration across agricultural landscapes and preserve/increase biodiversity [18]. Once an integral part of the historical landscape, such linear vegetation elements [19] help fragment open landscapes [20,21], improve habitat connectedness [22] and reduce erosion [23,24]. Over the medium and long-term, such linear woodland structures can also improve and/or restore soils, particularly alongside extensive agrosystems, by reducing bulk density, soil compaction and surface water content (waterlogging) and increasing organic matter content, mineral nitrogen (N) concentration, hydraulic conductivity [5,25].

Presence of woodland vegetation can also increase the activity of different forms of edaphon, leading to more effective nutrient and energy utilisation within the soil system. Biological activity in the soil is commonly expressed as either basal (soil) respiration [6,26], or respiration induced by different kinds of substrate, such as C (represented by glucose C₆H₁₂O₆) or N and S (represented by ammonium sulphate (NH_4_)_2_SO_4_) [[27], [28], [29]]. In this way, over fertilisation [[30], [31], [32]] may accelerate soil respiration by boosting microbial activity [29] but also reduce the efficiency of nutrient uptake from raw organic matter. While maintenance of soil “biological health” and ecological functioning (i.e. provision of optimal amounts and proportions of nutrients) [17] is key to safe food production and sustainable landscape management, this need is still insufficiently perceived by agronomists [33,34].

Biocorridors also play a significant role in improving both the quantity of organic matter and the form it takes, with leaf litter from forested biocorridors helping restore humification processes and increasing carbon sink (C-sink) levels compared with open agricultural land [[35], [36], [37], [38]]. Furthermore, humic substances are an important stabilising component, supporting soil structure, water retention capacity [39,40], nutrient sorption and biological activity, with levels of ca. 2 % considered the minimum for soil health.

While much of the information outlined above has been confirmed through small-scale studies and laboratory-based testing, there have been relatively few field-based studies comparing large established biocorridors with open agricultural land, allowing for robust generalisations of biocorridor impacts on soil quality and functioning. Here, we compare the soils of long-established biocorridors (30- and 60-years) with local cultivated farming soils using a wide spectrum of physical, hydrophysical, chemical and biological tools to assess whether there are indeed noticeable differences in soil properties after 30 and 60 years. We hypothesise that biocorridor soils will display reduced compaction and significantly improved pore structure, bulk density, water retention, microbial activity and nutrient availability over adjacent farmland soils due to long-term humification processes and natural sequestration of carbon.

## 2 Material and methods

### 2.1 Study site

**Fig. 1:**
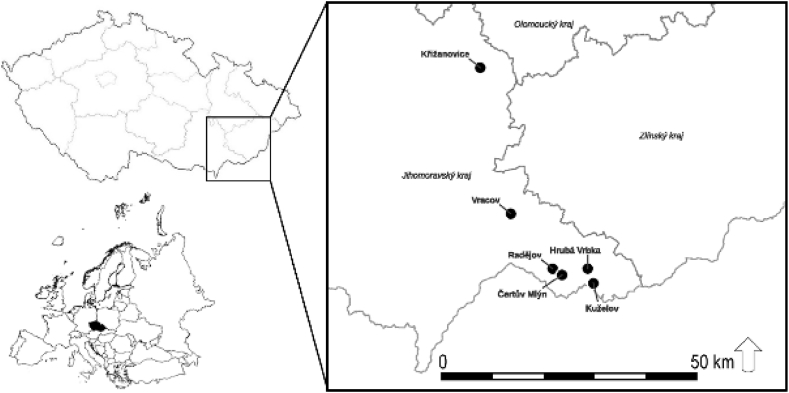
Localisation of the six biocorridor/farmland study plots.

For this study, soil samples were collected from six 40–60 m wide biocorridors, each of varying length and altitude, and from nearby farmland under modern agricultural management (up to 300 m on all sides of the biocorridor) in the Outer Western Carpathian Benchlands (Czech Republic; Fig. 1, Table 1). Each biocorridor was made up of a continuous stand of first-generation mixed forest, varying in age from either 30-years (Vracov, Křižanovice and Radějov) or 60-years (Kuželov, Čertův Mlýn and Hrubá Vrbka). Tree species composition was diverse and typical of low altitudes, including such species as field maple (*Acer campestre* L.), wild cherry (*Prunus avium* (L.) L.), small-leaved lime (*Tilia cordata* Mill.), oaks (*Quercus* sp.) and guelder rose (*Viburnum opulus* L.). The farmland was sown with common annual or biennial crops such as sweet corn (*Zea mays* L.) and wheat (*Triticum aestivum* L.) (Vracov, Křižanovice, Radějov), or left as meadow and grassland (Čertův Mlýn, Hrubá Vrbka, Kuželov). In all cases, the cultivated soil layer (plough layer) ranged from 10 to 25 cm.

**Table 1:** Position and base characteristics of the six biocorridor/farmland study sites in the Outer Western Carpathian Benchland, Czech Republic (see Fig. 1).

Site	Altitude [m a.s.l.]	Coordinates (N)	Coordinates (E)	Age [yrs.]	Biocorridor length [m]	Topography
Vracov	200–215	48°59′8.666"	17°11′2.628"	30	1200	Flat
Křižanovice	260–270	49°17′54.637"	17°1′57.348"	30	640	Flat
Radějov	225–250	48°52′28.464"	17°20′27.328"	30	1100	Undulating
Kuželov	370–440	48°51′4.512"	17°28′52.991"	60	200	Undulating
Čertův Mlýn	320–340	48°51′45.769"	17°22′27.892"	60	800	Undulating
Hrubá Vrbka	285–340	48°52′56.291"	17°27′28.888"	60	1400	Undulating

The climate in the study region is classified as warm to moderately warm [41,42] (Table 2). Geologically, most of the plots are classed under the “clayey” and/or “Karpaty Mts. calcareous flysch” geological units, with an admixture of fine-grain calcareous sandstone. However, Vracov was characterised by an admixture of wind-blown sand, while Křižanovice was classed under aeolian quaternary sediment (loess) (Table 2). In all cases, the soil-forming substrate comprised moderately rich to rich polygenetic loams of differing texture.

**Table 2:** Site characteristics of the six biocorridor/farmland study plots. Position: S = south; C = centre; N = north; Texture class: S = sand; r = silt; c = clay, L = loam); TOC = total organic carbon; * climatic region according to Quitt [41].

Site	Soil forming substrate	Climatic region*	Position	Soil taxon. unit	Soil texture class (30/60 cm)	TOC [%] (30/60 cm)	CaCO_3_ [%] (30/60 cm)	pH/KCl (30 cm)
Vracov	Fluvio-lacustrine sediment mixture of silt and sand	W4	S	Brunic Arenosol, (Protostagnic)	sL/S	2.30/2.28	0.10/0.13	4.5
			C	Luvic Cambisol, (Protocalcic)	L/lS	2.50/2.16	0.12/0.13	5.0
			N	Haplic Luvisol (Protocalcic)	L/sL	2.12/2.45	0.13/0.13	5.7
Křižanovice	quaternary sediments (loess, loess loam)	W2 (MW11)	S	Haplic Chernozem	rL/L	3.91/3.62	0.15/0.15	6.4
			C	Luvic Chernozem	rL/L	3.09/3.26	0.38/0.24	5.4
			N	Luvic Chernozem (Protostagnic)	rL/cL	3.27/2.65	0.10/0.10	6.3
Radějov	calcareous flysch (dominance of fine-grain calcareous sandstone), calcium claystone admixture	W2	S	Mollic Leptosol (Clayic, Protocalcic)	rcL/L	3.9/3.70	0.40/0.50	6.5
			C	Mollic Calcic Leptosol (Clayic, Protocalcic)	cL/C	3.29/2.56	0.10/0.13	6.4
			N	Cambic Calcaric Leptosol (Protocalcic)	cL/L	3.83/1.27	1.9/20.50	6.9
Kuželov	calcareous flysch (dominance of calcium claystone; minuscule fine-grain calcareous sandstone)	W2	S	Calcaric Leptosol (Clayic, Protostagnic)	rcL/cL	3.06/2.46	9.00/24.30	6.0
			C	Haplic Luvisol (Clayic, Protostagnic)	rcL/L	2.57/1.99	0.08/0.05	4.5
			N	Cambic Calcaric Leptosol	rcL/L	2.22/3.61	4.70/29.00	7.0
Čertův Mlýn	calcareous flysch (mixture of claystone of fine-grain calcareous sandstone)	W2	S	Calcaric Leptosol (Protocalcic, Protostagnic)	cL/lS	4.36/2.49	0.08/0.10	5.2
			C	Stagnic Luvic Phaeozem (Hyperhumic)	L/sL	3.36/3.09	0.10/0.10	5.5
			N	Stagnic Luvic Phaeozem (Hyperhumic)	cL/cL	3.74/3.06	0.05/0.10	4.7
Hrubá Vrbka	calcareous flysch (dominance of calcium claystone; minuscule fine-grain calcareous sandstone)	W2	S	Mollic Calcaric Leptosol (Clayic)	rcL/L	2.86/2.36	11.85/30.40	6.9
			C	Calcic Pelic Vertisol (Mollic)	rC/sC	3.49/3.07	0.25/17.65	6.8
			N	Mollic Calcaric Leptosol (Clayic)	rC/C	4.04/3.14	0.29/1.96	6.8

Unlike the farmland, where repeated cultivation has led to a homogenised plough layer, the biocorridor soil surfaces were typified by humification processes and formation of organic horizons. The humic layer was typically a mull [42]. Localised semi-hydromorphism was also recorded, especially at clay-rich sites.

### 2.2 Soil sampling

Soil pits were dug in both biocorridors and farmland to determine diagnostic horizons and soil taxonomic units, the latter being based on the World Reference Base [43]. In each biocorridor, samples were taken from three sampling positions along longitudinal axes, i.e. ‘North’, 60 m from the northern edge; ‘South’, 60 m far from the southern edge, and Centre, along the middle length of the biocorridor. Farmland sampling sites were situated perpendicularly to the biocorridor longitudinal axes and 20 m from the biocorridor edge. In total, 36 soil pits were excavated (18 in the biocorridors and 18 paired sites in neighbouring farmland).

From each soil pit we collected two types of sample. First, a mixed sample was taken from 0 to 30 cm in three repetitions, giving 108 mixed samples in total. Second, an undisturbed (core) sample was taken from 30 ± 5 cm and 60 ± 5 cm using a 100 cm^3^ iron ring, with three repetitions for each depth giving 216 core samples in total. The mixed soil samples were used to assess chemical and biological soil properties of 0–30 cm, while undisturbed samples were used to assess physical and hydrophysical properties and carbon stock in the depth of 30 ± 5 cm and subsoil 60 ± 5 cm).

### 2.3 Laboratory analysis

Each disturbed sample was divided into two portions comprising fine earth, which was then air dried, and a ‘fresh’ sample, which was stored at 4 °C. Both portions were passed through a 2 mm sieve prior to further analysis. The fine earth was used to assess: i) soil reaction as active (pH/H_2_O) or potential exchangeable (pH/KCl) in a 1:2.5 w/v suspension of water or 0.2 M KCl, respectively; ii) cation exchange capacity (*CEC* [mmol_+_ kg^−1^]), using the summation method [44]; iii); available nutrients (Ca, Mg, K [mg kg^−1^]) from Mehlich II leachate [45], along with the Mg/K ratio; iv); P content [mg kg^−1^], determined spectrophotometrically in a solution of ascorbic acid (C_6_H_8_O_6_), sulphuric acid (H_2_SO_4_) and antimony (Sb^3+^); and v) base saturation (*BS*), calculated using the equation *BS*=(Ca^2+^ + Mg^2+^ + K^+^)/*CEC* [%]; vi) hydrogen cation (H^+^) concentration [mmol_+_ kg^−1^], using dual pH measurements [46].

Both undisturbed and mixed samples were used to determine: i) total organic carbon (TOC; [%]), assessed spectrophotometrically in chromosulphuric acid (Cr₂K₂O₇·H₂O₄S) [47]; ii) total N (Nt [%]), assessed using the Kjeldahl method [48], and the C/N ratio, calculated from the mixed sample assessment; iii); carbonates (CaCO_3_), assessed quantitatively through carbonate decomposition with diluted (12.5 %) hydrochloric acid (HCl); iv) concentration of humic substances [%], determined as humic acid carbon (C-HA), fulvic acid carbon (C-FA), the C-HA/C-FA ratio (HA/FA) and sum (C-THS) [49]; v) ammonia N (N–NH_4_^+^) concentration [mg 100 g^−1^], assessed in H_2_O using a solution of sodium salicylate (C_7_H_5_NaO_3_), trisodium citrate dihydrate (C_6_H_5_Na_3_O_7_.2H_2_O), sodium nitroprusside (C_5_FeN_6_Na_2_O) and an alkaline solution (sodium hydroxide (NaOH) + sodium dichloroisocyanurate (C_3_Cl_2_N_3_NaO_3_)) as the colouring agent; and vi) nitrate N (N–NO_3_^-^) concentration [mg 100 g^−1^], ascertained in a solution of sulphuric acid and sodium salicylate [50].

Fresh samples were used to assess respiration [mg CO_2_ 100 g^−1^ h^−1^], determined in different forms depending on the substrate added to stimulate soil microbial activity after 20 h incubation, i.e. basal respiration (*RB*), after addition of 2 ml H_2_O, and substrate-induced respiration (*SIR*), after addition of 2 ml of 20 % glucose solution (*RG*), 2 % ammonium sulphate solution (*RN*), or their combination at a ratio of 1:1 (*RNG*) [51]. Respiration was then measured as the amount of CO_2_ produced following titration of sodium hydroxide by hydrochloric acid. Soil microbiota metabolism and the effectiveness of organic matter consumption were then calculated by computing the ratios of the different forms of respiration, i.e. *RN/RB*; *RG/RB*; *RG/RN* and *(RNG/RG)/(RN/RB)* [52,53].

Undisturbed samples were used to assess bulk density (*ρ*_*d*_; [g cm^−3^]) and soil hydrolimits [% vol.] [54,55]. Soil hydrolimits were determined by weighing the undisturbed sample at different water saturation states. First, the soil sample was weighed fresh and then placed in a suction apparatus composed of a plate packed with filter paper and flooded to 5 mm below the upper edge. After full saturation (after 24 h), the sample was repeatedly placed on three dry filter papers and weighed at intervals of 30 min, 2 h, 24 h and two weeks (air-dried), after which the samples were oven-dried at 105 °C. Throughout the procedure (aside from oven drying), the samples were covered with a watch-glass to avoid evaporation. Hydrolimits were then expressed as water holding capacity at different levels of water saturation, i.e. saturated water content (*Θ*_*S*_), where a sample is fully saturated after 24 h suction, and gravitational water (*Θ*_*G*_), maximum capillary capacity (*Θ*_*MCC*_) and retention water capacity (*Θ*_*RWC*_) following 0.5, 2 and 24 h water suction at *Θ*_*S*_, respectively. The permanent wilting point (*Θ*_*PWP*_) was assessed using the equation *Θ*_*PWP*_ = *Vhk*, where *Vh* is hygroscopic moisture (calculated as the air-dry water content multiplied by 1.8) and *k* is the empirical constant given by the soil texture (i.e. 2 for clay, 2.5 for sandy-clay loam, clay-loam and silty-clay loam, and 3 for sand, loamy-sand, sandy-loam, loam, silt-loam and silt). After sieving and air-drying the undisturbed samples, specific density (*ρ*_*s*_; [g cm^−3^]) was assessed using Gay-Lussac pycnometer soil sample boiling [44], and soil texture using the pipette analysis for assessing clay (<0.002 mm), silt (0.002–0.05 mm) and sand (0.05–2 mm) [%] fractions. Soil porosity (*P*) was then calculated using equation *P* = (*ρ*_*s*_ – *ρ*_*d*_)/*ρ*_*s*_ 100 [% vol.]; available water capacity (*AWC*) using *AWC* = *Θ*_*RWC*_ – *Θ*_*PWP*_ [mm] for a 200 mm soil column; minimum aeration capacity (*A*_*MCC*_) using *A*_*MCC*_ = *P* – *Θ*_*MCC*_ [% vol.]; non-capillary pores (*P*_*G*_) using *P*_*G*_ = *Θ*_*s*_ – *Θ*_*G*_ [% vol.] and semi-capillary pores (*P*_*SC*_) using *P*_*SC*_ = *Θ*_*G*_ – *Θ*_*RWC*_ [% vol.], with capillary pores (*P*_*C*_) expressed as *Θ*_*RWC*_ and the *P*_*C*_:*P*_*G*_ ratio.

TOC-stock [g m^−2^ 0.1 m^-1^] was calculated from undisturbed sample *ρ*_*d*_ and TOC and expressed for soils at 30 ± 5 and 60 ± 5 cm depth using the equation TOC-stock = TOC/100 · *ρ*_*d*_ · *a* · *H*, where *a* is an area of 1 m^2^ and *H* is soil depth of 0.1 m.

### 2.4 Statistical analysis

Owing to the hierarchical structure of the sampling design and its flexibility, data processing was based on Bayesian multilevel (mixed) models, in which site and sampling position were treated as random effects and management type (biocorridor/farmland) as a fixed effect. As the data for most soil properties were more-or-less normally distributed, a normalised distribution was assumed in these cases; however, P, Mg, Ca, K and CaCO_3_ all showed a left-skewed distribution, hence gamma distribution (with the natural logarithm link function) was used when assessing these parameters. For physical and hydrophysical properties, the effect of management type was assessed separately at 30 cm and 60 cm depth. Statistical model parameters were estimated using the Markov Chain Monte Carlo (MCMC) method, as implemented in the Stan program [56].

All analyses were undertaken using the ‘brms’ package v. 2.14.4 [57,58] in the R software environment [59], using the brms default priors and four chains with 5000 iterations per chain (including 2000 as warmup iterations). For each soil property, we report the estimated average value (median and posterior distribution) for farmland, along with 89 % credible intervals (CI), and the estimated difference (median of posterior distribution) between biocorridor and farmland, with 89 % credible intervals and probability of direction (pd), i.e. the probability that an effect goes in a particular direction (positive/negative). For pd, values range from 0.5 to 1, where 0.5 indicates high uncertainty in the direction of effect, and therefore in the effect itself, while 1 indicates high certainty in the effect direction. In all cases, conditional R^2^ reflects variability explained by the whole model (including random effects) and marginal R^2^ reflects variability explained by fixed effects only, i.e. management type.

To obtain an overview of relationships between soil properties, correlation matrices were constructed separately for the biocorridor and farmland data subsets using the ‘corrplot’ package in R [60]. The statistical significance of correlation coefficients was expressed with a critical value of 0.263 (n = 54).

## 3 Results

### 3.1 Physical and hydrophysical properties

**Fig. 2:**
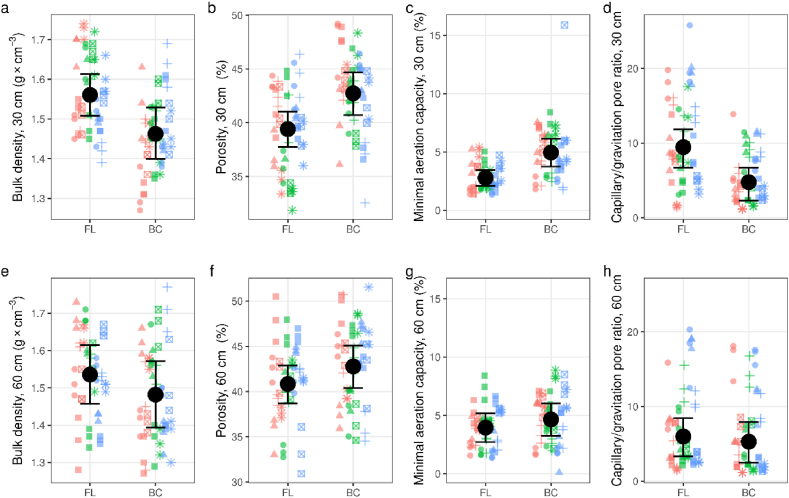
Graphical outputs for biocorridor (BC) and farmland (FL) soil properties statistical processing at different soil depths. a–d = 30 cm depth; e–h = 60 cm depth; points = different biocorridors (circle = Čertův Mlýn; triangle = Hrubá Vrbka; square = Křižanovice; plus = Kuželov; square cross = Radějov; asterisk = Vracov); red, green and blue refer to samples south, centre and north of the biocorridor, respectively; Error bars = 89 % credible intervals.

Aside from saturated water content (*Θ*_*S*_), which was significantly higher in biocorridors at 30 cm, water retention capacity soil parameters (hydrolimits) for biocorridor and farmland soils showed no significant differences (Table 3, Fig. 2). However, though the differences were not significant, water retention capacity values (*Θ*_*MCC*_, *Θ*_*RWC*_ and *Θ*_*PWP*_) in farmland soil did decrease slightly with depth, and increase with depth in biocorridor soils (Table 3, Fig. 2).

**Table 3:** Hydrophysical soil properties and TOC-stock across biocorridor and farmland plots at different sampling depths. BC = biocorridor; FL = farmland; CI = 89 % credible intervals; pd = probability that an effect goes in a particular direction (significant values (p < 0.05) in italic); *Θ*_*S*_ = saturated water content; *Θ*_*G*_ = gravitational water content; *Θ*_*MCC*_ = maximum capillary capacity; *Θ*_*RWC*_ = retention water capacity; *Θ*_*PWP*_ = permanent wilting point; *ρ*_*d*_ = bulk density; *P* = porosity; *AWC* = available water capacity; *A*_*MCC*_ = minimum aeration capacity; *P*_*C*_ = capillary pores; *P*_*G*_ = non-capillary pores; TOC-stock = total organic carbon stock.

Parameter	Units	Soil depth	Estimated average value		Difference between BC and FL			conditional R^2^	marginal R^2^
		[cm]	BC	FL	Point estimate	(CI)	pd		
***Θ***_***S***_	**% vol.**	*30*	*42.7*	*41.0*	*1.78*	*(0.20, 3.37)*	*0.962*	*0.790*	*0.041*
		60	43.1	41.9	1.27	(-0.37, 2.89)	0.897	0.851	0.019
***Θ***_***G***_		30	39.8	39.3	0.5	(-1.14, 2.13)	0.705	0.810	0.007
		60	40.4	39.6	0.83	(-0.80, 2.45)	0.805	0.873	0.009
***Θ***_***MCC***_		30	37.7	38.1	−0.4	(-1.85, 1.06)	0.68	0.872	0.005
		60	38.3	37.8	0.54	(-1.13, 2.20)	0.709	0.898	0.006
***Θ***_***RWC***_		30	34.0	35.5	−1.51	(-3.12, 0.08)	0.937	0.903	0.020
		60	34.1	34.2	−0.07	(-1.61, 1.49)	0.525	0.913	0.003
***Θ***_***PWP***_		30	26.2	27.0	−2.3	(-4.95, 0.37)	0.921	0.915	0.023
		60	27.5	26.7	0.78	(-1.85, 3.42)	0.699	0.941	0.005
***ρd***	**g cm**^**−**^**^3^**	*30*	*1.46*	*1.56*	*−0.1*	*(-0.17, -0.03)*	*0.976*	*0.816*	*0.221*
		*60*	*1.48*	*1.54*	*−0.05*	*(-0.1, 0.0)*	*0.955*	*0.854*	*0.05*
***P***	**% vol.**	*30*	*42.7*	*39.4*	*3.3*	*(1.12, 5.44)*	*0.989*	*0.800*	*0.176*
		*60*	*42.8*	*40.8*	*1.95*	*(0.23, 3.66)*	*0.961*	*0.844*	*0.047*
***AWC***	**mm**	30	16.6	15.0	1.57	(-2.47, 5.59)	0.745	0.792	0.016
		60	13.7	15.8	−2.08	(-6.18, 1.98)	0.802	0.805	0.020
***A***_***MCC***_	**% vol.**	*30*	*4.96*	*2.77*	*2.19*	*(1.26, 3.10)*	*0.998*	*0.668*	*0.268*
		60	4.66	3.95	0.71	(-0.07, 1.48)	0.930	0.856	0.035
***P***_***C***_***: P***_***G***_	**-**	*30*	*4.67*	*9.39*	*−4.71*	*(-6.44, -3.00)*	*1.000*	*0.794*	*0.207*
		60	5.27	5.96	−0.69	(-2.10, 0.65)	0.797	0.899	0.005
**TOC-stock**	**g m**^**−**^**^2^ 10 cm**^**−**^**^1^**	30	4656	4905	−249.49	(-964, 417)	0.734	0.863	0.019
		*60*	*3999*	*3392*	*607.67*	*(40.5, 1181.3)*	*0.955*	*0.857*	*0.083*

Both biocorridor and farmland soil bulk density (*ρ*_*d*_) (Fig. 2a and e) and porosity (*P*) (Fig. 2b and f) differed significantly, especially at 30 cm, with biocorridor soils having higher porosity and lower bulk density, indicating lower compaction, and farmland soils having more capillary pores (*ρ*_*c*_) (particularly at 30 cm), suggesting an increased water retention (Table 3, Fig. 2). Farmland soils also showed an increased permanent wilting point (*Θ*_*PWP*_; especially at 30 cm; Table 3), further indicating lower physiological water availability and aeration (with very narrow data variability (Fig. 2c and g)) than biocorridor soils. In comparison, related *AWC* values were not significantly different, with values ranging between ca. 13.5 and 17 mm per 200 column of soil in both farmland and biocorridors (Table 3).

While there was no significant difference in the capillary (*P*_*c*_) and non-capillary (*P*_*g*_) pore ratio at 60 cm (Fig. 2h), where capillary pores represented up to 80 % of total porosity (*P*), ratios were significantly lower in biocorridors at 30 cm (Fig. 2d), suggesting more favourable hydrophysical conditions (Table 3, Fig. 2). The resulting lower minimum aeration capacity (*A*_*MCC*_) values suggest a deficiency in non-capillary pores, especially in farmland soils. At all sites, homogenous minimum aeration capacities resulted in narrow credible intervals, especially in the topsoil, despite the significant differences between biocorridor and farmland soils (Fig. 2).

### 3.2 Carbon content and sequestration

There was no significant difference in either TOC or inorganic carbon content (CaCO_3_) at different depths in biocorridor or farmland soils (Table 3, Table 4). TOC-stock was higher at 30 cm in both biocorridor and farmland soils; and though farmland values tended to be slightly higher at 30 cm, biocorridor values were significantly higher at 60 cm, with values around 600 g m^−2^ 10 cm^−1^ higher than in farmland (Table 3, Table 4). TOC concentrations were characterised by relatively high variability, especially in biocorridor soils, with the main source of variability being locality (Supplementary materials 1).

**Table 4:** Chemical soil properties across research plots for biocorridors and farmland at 0–30 cm. BC = biocorridor; FL = farmland; CI = 89 % credible intervals; pd = probability that an effect goes in a particular direction (significant values (p < 0.05) in italic); pH/H_2_O = active soil reaction; pH/KCl = potential exchangeable soil reaction; P, Mg, Ca, K = available nutrient; *CEC* = cation exchangeable capacity; TOC = total organic carbon; N_t_ = total nitrogen; C-HA = humic acid carbon; C-FA = fulvic acid carbon.

Parameter	Units	Estimated average value		Difference between BC and FL			conditional R^2^	marginal R^2^
		BC	FL	Point estimate	(CI)	pd		
pH**/H**_**2**_**O**	**-**	6.57	6.71	−0.14	(-0.51, 0.22)	0.766	0.947	0.018
pH**/KCl**		5.84	5.94	−0.10	(-0.54, 0.35)	0.645	0.934	0.013
**P**	**mg kg**^**−**^**^1^**	*17.2*	*25.8*	*−8.57*	*(-15.79, -2.98)*	*0.985*	*0.977*	*0.032*
**Mg**		*301.6*	*263.5*	*38.14*	*(1.02, 77.86)*	*0.949*	*0.938*	*0.055*
**Ca**		3745.2	3303.0	442.10	(-189.2, 1195.5)	0.884	0.984	0.005
**K**		203.6	217.4	−13.77	(-79.06, 60.7)	0.707	0.981	0.009
***CEC***	**mmol** + **kg**^**−**^**^1^**	260.1	226.9	33.15	(-2.97, 74.40)	0.933	0.986	0.011
**TOC**	**%**	2.69	2.22	0.47	(-0.02, 0.94)	0.939	0.978	0.045
**Nt**		0.23	0.20	0.03	(-0.01, 0.06)	0.908	0.963	0.057
**C/N**	**-**	10.9	10.4	0.51	(-0.14, 1.18)	0.902	0.929	0.004
**CaCO**_**3**_	**%**	0.97	0.73	0.23	(-0.20, 0.78)	0.753	0.98	0
**C-HA**		0.23	0.22	0.02	(-0.04, 0.07)	0.687	0.932	0.012
**C-FA**		*0.32*	*0.24*	*0.08*	*(0.01, 0.16)*	*0.961*	*0.916*	*0.144*
**C-HA/C-FA**	**-**	*0.75*	*0.90*	*−0.15*	*(-0.28, -0.02)*	*0.962*	*0.859*	*0.066*

### 3.3 Chemical properties

All soils were characterised by a relatively neutral soil reaction, with no significant differences between biocorridor and farmland (Table 4). This corresponds with the high degree of base saturation, with saturation of the sorption complex mainly resulting from a high concentration of calcium (Supplementary material 1). Again, in both soil types, total cation exchange capacity increased with TOC, though more so in biocorridors (Fig. 5). Nutrient availability varied between soil types, with no significant difference in calcium and potassium contents but significantly higher phosphorus concentrations in farmland and significantly higher magnesium concentrations in biocorridors (Table 4). There were no significant differences in either total nitrogen content or the C/N ratio between soil types; and while there was no significant difference in C-HA, C-FA concentrations were significantly higher in biocorridor soils, resulting in a lowered C-HA/C-FA ratio (Table 4).

### 3.4 Mineral nitrogen content and soil respiration

**Fig. 3:**
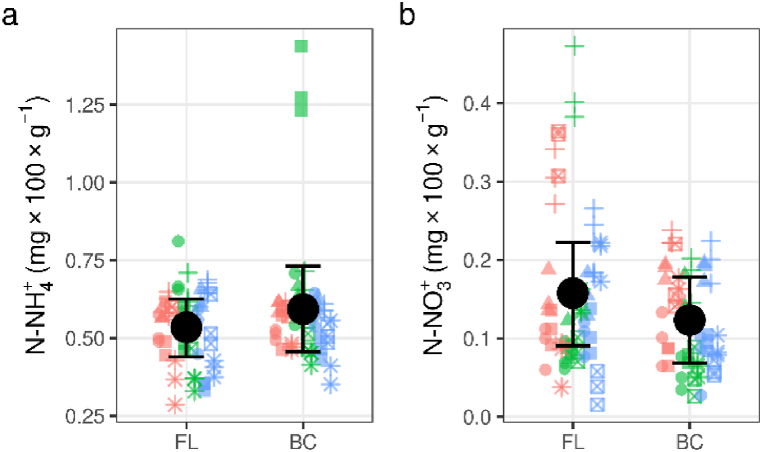
Mineral nitrogen content in biocorridor (BC) and farmland (FL) soils. a = (N–NH_4_^+^) nitrogen as ammonia; b = (N–NO_3_^-^) nitrogen as nitrate; points = different biocorridors (circle = Čertův Mlýn; triangle = Hrubá Vrbka; square = Křižanovice; plus = Kuželov; square cross = Radějov; asterisk = Vracov); red, green and blue refer to samples south, centre and north of the biocorridor, respectively; error bars are 89 % credible intervals.

**Fig. 4:**
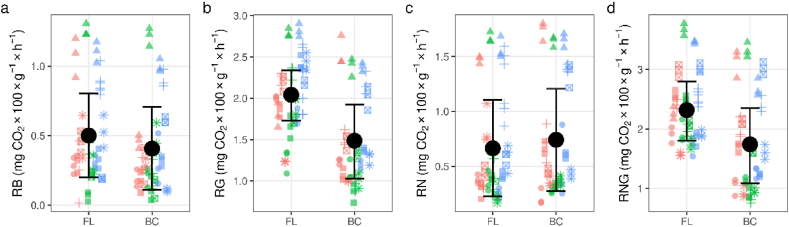
Soil respiration activity in biocorridor (BC) and farmland (FL) soils. a = (*RB*) basal respiration; b = (*RG*) glucose-induced respiration; c = (*RN*) ammonium sulphate-induced respiration; d = d = (*RNG*) ammonium sulphate: glucose in 1 : 1 ratio-induced respiration, respectively; points = different biocorridors (circle = Čertův Mlýn; triangle = Hrubá Vrbka; square = Křižanovice; plus = Kuželov; square cross = Radějov; asterisk = Vracov); red, green and blue refer to samples south, centre and north of the biocorridor, respectively; error bars are 89 % credible intervals.

Aside from biocorridor soil at Křižanovice, where ammonia nitrogen was ca. two times higher, biocorridor and farmland soils at most sites displayed similar concentrations of available mineral nitrogen forms (Table 5; Fig. 3a and b; see Supplementary material 2 for values from each site). Respiration activity varied over a wide range and differed within localities, especially at Hrubá Vrbka, where values were several times higher than at other sites (Fig. 4). *RN* did not show significant differences when comparing FL and BC (Fig. 4c). In biocorridors, *RG* (Fig. 4b) and *RNG* (Fig. 4d) were significantly lower (ca 27 % and 25 %, respectively), as were the *RG/RN* and *RNG/RG/RNB* ratios (ca. 42 % and 31 %, respectively) (Table 3), indicating higher physiological N utilisation in farmland and a higher proportion of difficult to utilise N forms in biocorridor soils.

**Table 5:** Mineral nitrogen content and soil respiration across research plots for biocorridor (BC) and farmland (FL) soils. BC = biocorridor; FL = farmland; CI = 89 % credible intervals; pd = probability that an effect goes in a particular direction (significant values (p < 0.05) in italic); N–NH_4_^+^ = nitrogen as ammonia; N–NO_3_^-^ = nitrogen as nitrate; *RB* = basal respiration; *RN, RG* and *RNG* = respiration induced by ammonium sulphate, glucose and ammonium sulphate:glucose at 1:1 ratios, respectively.

parameter	units	Estimated average value		Difference between BC and FL			conditional R^2^	marginal R^2^
		BC	FL	Point estimate	(CI)	pd		
**N–NH**_**4**_^**+**^	**mg N–NH**_**4**_+ **100g**^**−**^**^1^**	0.59	0.53	0.06	(-0.07, 0.18)	0.812	0.893	0.046
**N–NO**_**3**_^-^	**mg N–NO**_**3**_- **100g**^**−**^**^1^**	0.12	0.16	−0.03	(-0.08, 0.01)	0.897	0.903	0.039
***RB***	**mg CO**_**2**_**100 g**^**−**^**^1^** **h**^**−**^**^1^**	0.41	0.50	−0.11	(-0.23, 0.02)	0.922	0.936	0.025
***RG***		*1.48*	*2.04*	*−0.56*	*(-0.9, -0.23)*	*0.990*	*0.943*	*0.243*
***RN***		0.74	0.67	0.06	(-0.1, 0.23)	0.746	0.958	0.006
***RNG***		*1.73*	*2.31*	*−0.57*	*(-0.88, -0.27)*	*0.993*	*0.979*	*0.139*
***RN/RB***	**-**	2.37	1.93	0.44	(-0.46, 1.32)	0.793	0.237	0.01
***RG/RB***	**-**	4.53	7.48	−2.95	(-7.24, 1.32)	0.867	0.117	0.012
***RG/RN***	**-**	*2.54*	*4.35*	*−1.81*	*(-2.9, -0.75)*	*0.992*	*0.865*	*0.169*
***RNG/RB***	**-**	5.00	8.52	−3.52	(-8.58, 1.49)	0.871	0.151	0.012
***RNG/RG/RNB***	**-**	*0.66*	*0.96*	*−0.30*	*(-0.51, -0.09)*	*0.982*	*0.576*	*0.101*

**Fig. 5:**
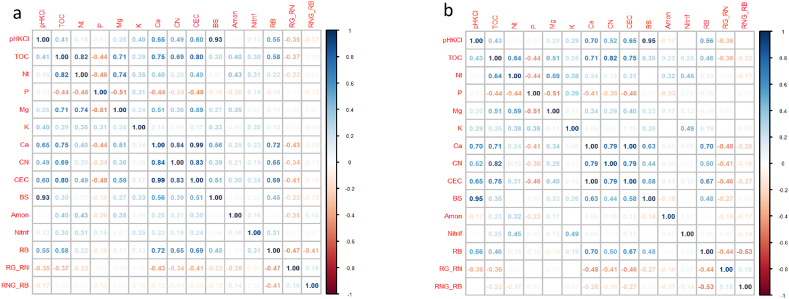
Correlation matrices for (a) biocorridor and (b) farmland soil properties, with Pearson's correlation coefficients shown. pHKCl = potential exchangeable soil reaction; TOC = total organic carbon; Nt = total nitrogen; P, Mg, K, Ca = available nutrient concentrations; CN=C/N ratio; CEC = cation exchange capacity; BS = base saturation; Amon = nitrogen as ammonia, Nitrif = nitrogen as nitrate; *RB* = basal respiration; *RG* = glucose-induced respiration; *RN* = ammonium sulphate-induced respiration.

In general, correlations were stronger for biocorridor soils (Fig. 5a) than farmland (Fig. 5b), particularly regarding the importance of soil organic carbon. In biocorridor soils, total organic carbon concentration was significantly correlated with total nitrogen, ammonia nitrogen and nitrate nitrogen contents; thus, while total organic carbon concentration was comparable in all soils, it played a greater role in nitrogen fixation in biocorridor soils. The strong correlation between ammonia nitrogen and total nitrogen contents in biocorridors indicates that ammonia nitrogen plays a stronger role in biocorridor soils, while the significant negative correlation between nitrate nitrogen and basal respiration rate indicates that soil microbiota play a more important role in biocorridor metabolic and physiological processes.

## 4 Discussion

### 4.1 Physical and hydrophysical properties

Soil textural composition plays a crucial role in defining the ecological limits given by edaphic environmental conditions. Soil development with flysch soil forming substrate leads to textural stratificxation of soil body that defines both localities and sampling sites within the localities. In our study, there were clear differences in soil quality between farmland soils under conventional agricultural management and those under biocorridors with long-established woodland. In general, farmland topsoils were typified by high (2 × ) capillary/non-capillary pore ratios, with the proportion of capillary pores higher in biocorridor soils, and the proportion of non-capillary pores higher in biocorridor soils leading to optimisation of soil aeration. Together, these indicate that farmland soils show increased dryness, reduced aeration, a heavier texture and higher compaction than lighter, more aerated forested biocorridor soils. Similar improvements in forested biocorridor soil conditions were noted by Holden et al. [25], the latter also noting that hedgerows improved soil properties by lowering topsoil bulk density, surface soil compaction and soil water content (i.e. a reduction in the degree of over-moistening), and increasing hydraulic conductivity, as also occurred in our study.

### 4.2 Carbon content and sequestration

In most cases, we observed higher concentrations (non-significant) of both organic and mineral carbon forms (TOC and CaCO_3_, respectively) at 0–30 and 30–60 cm, and lower TOC-stock levels (non-significant) at 30 cm but significantly higher levels (almost 18 %) at 60 cm, in biocorridor soils. Holden et al. [25] reported similar findings for soil organic carbon content under trees, the authors suggesting that differences with agricultural soils were attributable to disturbance by ploughing, which increased organic carbon decomposition rates. As a result, farmland tends to release CO_2_, while soil organic carbon under woodland is incorporated into deeper soil layers with decaying roots and other litter. Mayer [61], for example, suggested the increase in organic carbon under wooded biocorridors was most likely attributable to humification processes and natural sequestration of carbon in the soil over the period since biocorridor establishment, with a prerequisite for temporal development of total organic carbon concentration being the allocation of carbon in its various forms, including a gradual increase in the proportion of decaying wood [61,62]. In our case, biocorridor total organic carbon content was 0.3–1.3 % higher than in farmland, with highest values in trophically rich pelic soils on carbonate-silicate substrates, especially the Mollic Leptosols (Clayic) at Hrubá Vrbka, which were overshadowed by a small-leaved lime tree with high litter production. In contrast, the farmland reference sites displayed much higher variability, with organic carbon concentrations tending to reflect specific management practices and not necessarily intra-soil processes [63]. Owing to their potential role as C-sinks, some recent studies have suggested that biocorridors and hedgerows represent good candidates as climate-change mitigation tools [25,61,[63], [64], [65]]. Drexler et al. [64], for example, demonstrated the effectivity of hedgerows as C-sinks in a large meta-analysis, showing that they were 30 % more effective at storing soil organic carbon than farmland. Similarly, Biffi et al. [65] recorded a 31 % increase in soil carbon sequestration in hedgerows compared to grassland, whether on sandstone (acid) or limestone (basic) beds.

### 4.3 Chemical properties

In a similar manner to our study, Valverde-Asenjo et al. [66] detected a significant increase in soil carbon and nitrogen 20 years after a vineyard was abandoned and became overgrown. One possible cause for this was highlighted by Heydari et al. [67], who, in their studies of perennial land in a semiarid region of Iran, demonstrated changes in soil chemistry, including an increase in N, due to acidification processes associated with carbonate [CO_3_^2^⁻] leaching. In our own study, local soil conditions (including pH; see Table 2) varied greatly between biocorridor and farmland sites, due in large part to soil forming substrate chemistry. Heavy-textured (clayey) soils, for example, increase the soil's buffering capacity, and this could have a negative impact on the ability of biocorridor tree cover (or the time since change in land use) to improve soil conditions. Differences in vegetation cover associated with differing soil chemistry have also been reported by Sagheb-Talebi et al. [68], for carbonate region of Iran, and Binkley and Fisher [69], who showed that tree cover resulted in carbonate leaching due to natural acidification process, though this only occurred over longer periods. Interestingly, our data showed a slight increase in carbonate levels in biocorridor subsoils (60 cm), possibly related to natural leaching from the topsoil. On the other hand, topsoil carbonate leaching could also be related to soil protonation resulting from converting farmland to ‘forest’. In such cases, production of humic substances during the decomposition of decaying organic matter results in increased C-FA production and a lowered C-HA/C-FA ratio [70], a process also observed in the present study (see Table 4).

There are several possible causes of the significantly lower phosphorus levels recorded in biocorridor soil in our study, including the use of different leachates during soil analysis [25], bonding of phosphorus with calcium in alkaline environments [71] and differences in soil textural size composition at different sites [72]. Based on both the data collected and our own local knowledge of the study sites’ recent history, the higher P levels recorded on farmland are almost certainly the result of external addition as fertiliser, with the lower levels in biocorridor soils potentially reduced further through leaching processes related to tree cover (see above).

Presence of calcium has the potential to stabilise soil through the formation of aggregates, mitigation of acidification and immobilisation of risk elements [73], while magnesium plays a key role in maintaining plant physiological processes [[74], [75], [76]]. However, magnesium in particular is often deficient in soils, with subsequent negative impacts on plant nutrition and tolerance to salinity [77]. At our study localities, calcium and magnesium contents were both recorded at high to supra-optimal levels [78], and thus there appears to be no potential risk of deficiency. On the other hand, such high levels could potentially result in antagonism with K^+^ ions, thereby reducing potassium availability, a highly important nutrient as regards plant resistance to drought [79] and frost [80]. Our results, however, indicated relatively high nutrient levels in both biocorridor and, somewhat surprisingly, farmland soils (Table 4), suggesting they are being applied to farmland soils, presumably as part of a complex fertiliser admixture. Furthermore, the high sorption capacity recorded in the various farmland soils (Supplementary material 1) suggests a low risk of future leaching.

### 4.4 Mineral nitrogen and soil respiration

In our study, differences in the content of ammonia and nitrite forms of nitrogen in biocorridor and farmland soils were non-significant, which contrasts with the findings of Holden et al. [25], who recorded a three times increase in nitrate nitrogen, and reduced ammonia nitrogen, under hedgerows when compared with farmland soils. According to Thomas and Abbott [81], numerous factors could have affected both uptake and denitrification of nitrate nitrogen under hedgerows, including redox conditions, tree species composition, seasonality and legacy of fertiliser application.

While non-significant, nitrate nitrogen values were lower in our biocorridor soils, and while this may be partly related to site specifics such as soil chemistry and pH [82], other factors may also have played a role. Firstly, nitrification processes may have been limited by physical soil properties, e.g. high clay content and low *A*_*MCC*_, both of which lead to soil hydromorphisation and anaerobism [83]. Secondly, the local climate frequently lead to periods of drought during part of the growing season that can virtually eliminate soil biological activity. Thus, the concentration of mineral nitrogen forms can be taken to reflect both the state of biological activity during past growing season(s) and possible sources of nitrogen, i.e. the general conditions for the course of microbial transformation within the given ecosystem [84].

Typically, higher soil microbial respiration activity leads to more rapid nutrient turnover, especially in relation to decomposition processes [85]. Lower activity, typical of perennial vegetation, is related to increased system efficiency as regards soil TOC-stock, leading to reduced carbon emissions. In our study, biocorridor soils typically showed much lower microbial respiration activity than farmland, with glucose-induced (*RG*) values down by up to 27 %. The stronger positive correlations for basal respiration with C/N ratios in biocorridor soils can be construed as a sign of increased efficiency at utilising internal carbon sources. Conversely, the stronger correlations for total organic carbon content with C/N ratios in farmland are indicative of the artificial input of organic matter, typical of agrosystems. Similar outcomes were also observed in the studies of Baah-Acheamfour et al. [86], dealing with lowered CO_2_ emissions from soils below woody vegetation, and Ford et al. [26], who compared the effects of different vegetation types on soil.

The higher respiration activity recorded in our farmland soils (*RB* > 20 %, *RG* ca. 40 %) (Fig. 4a and b) is due to artificial soil enrichment and cultivation of the plough layer, i.e. through the addition of artificial fertilisers and animal manures and their distribution in the topsoil by ploughing. The resulting increase in depth of biological activity will have a positive effect on both plant root distribution and soil microbial activity, and thus nitrification processes, resulting in the increase in mineral N content (in the form of nitrate^−^) observed in this study. Conversely, incomplete redistribution of microbial activity within the topsoil may have been another contributing factor to the lower values in biocorridor soils, particularly as regards potential respiration (representing the energetic needs of microorganisms) [87]. Though generally low [88] at both site types, differences in the respiratory values observed between biocorridor and farmland soils could also be indicative of the length of time physiological process conditions have been suitable for soil microbiota [89], i.e. in our own study, this is likely to have occurred most frequently in the biocorridor root zone. Other potential management-related factors resulting in temporary or localised stimulation of microbial metabolism could include re-ploughing of post-harvest residues, denitrification processes under anaerobic conditions and/or in temperature changes at the end of the growing season [90].

The relatively low *RNG/RG/RNB* ratios in our biocorridor soils were indicative of the complex effect of soil properties on carbon and nitrogen availability, suggesting a reduction in both substance and nutrient flow more typical of forest environments than farmland soils. While the higher *RG/RN* values observed in farmland soils are a result of the addition of easily-available nitrogen sources, such as soluble fertilisers, nitrogen availability in biocorridor soil is instead reliant on N sources delivered by soil microbiota during the decomposition of raw organic matter. Likewise, the high *RNG/RB* ratio in farmland soils suggest that the resident soil microflora readily mineralise the organic matter added to the field and convert it into a form acceptable to plants.

## 5 Conclusion

There is growing evidence that provision of linear connective habitat features in open farmland, such as windbreaks and biocorridors, creates an ecological network that not only provides ecosystem services and improves landscape stability but also protects soil. In this study, we used a wide range of physical, hydrophysical, chemical and biological tools to compare soil/substrate characteristics of long-established (30- and 60-years) biocorridors soils with nearby cultivated farming soils. Before the biocorridors were established, the farmland soil was considered unfavourable due to a dominance of sedimentary clayey- and silty-clay substrates.

Our results clearly demonstrated the ameliorative effect of established tree cover, with improvements in biocorridor soil porosity, bulk density, aeration and TOC-stock, thereby confirming our hypothesis. While overall biological activity (expressed as either *BR* or *SIR*) remained relatively low in biocorridor soils due to the clay and/or silty clay soil conditions, metabolic quotients suggested that biocorridor microbiota were more effective at utilising organic matter as a C and N source, with both land use and presence of lighter-textured soils having a positive influence. Owing to their potential role as C-sinks, some recent studies have suggested that biocorridors and hedgerows (and woodlands) could represent good candidates as climate-change mitigation tools. However, there is still relatively little information available on the effect of biocorridors on different types of soil; consequently, we suggest follow-up studies that provide more detailed information on biological activity and soil functional diversity in different soil types.

## Data availability statement

Data will be made available on request.

## CRediT authorship contribution statement

**Aleš Kučera:** Conceptualization, Formal analysis, Validation, Writing – original draft, Writing – review & editing. **Dušan Vavříček:** Investigation, Methodology, Writing – original draft, Data curation. **Daniel Volařík:** Data curation, Formal analysis, Writing – original draft. **Pavel Samec:** Supervision, Writing – original draft. **Luboš Úradníček:** Funding acquisition, Methodology, Project administration, Writing – original draft.

## Declaration of competing interest

The authors declare that they have no known competing financial interests or personal relationships that could have appeared to influence the work reported in this paper.
