# Supplementary material for: Wooded biocorridors substantially improve soil properties in low-altitude rural benchlands
Source: Heliyon. 2024 Jan 17;10(2):e24381. doi: 10.1016/j.heliyon.2024.e24381 (PMC10835163; doi:10.1016/j.heliyon.2024.e24381)
Supplement: Multimedia component 1 [file mmc1.docx]

Supplementary material 1: Hydrophysical soil properties and soil carbon stock across research plots differently for the biocorridor (BC) and farm land (FL) in sampling depths. For abbreviations of soil parameters Methods section.

| **parameter** | **units** | **soil depth** | **mean** | | **sd** | | **median** | | **min** | | **max** | |
| --- | --- | --- | --- | --- | --- | --- | --- | --- | --- | --- | --- | --- |
|  |  | **[cm]** | **BC** | **FL** | **BC** | **FL** | **BC** | **FL** | **BC** | **FL** | **BC** | **FL** |
| Θ_S_ | **% vol.** | 30 | 42.9 | 41.1 | 3.8 | 4.9 | 44.0 | 41.2 | 33.7 | 30.1 | 51.8 | 49.7 |
|  |  | 60 | 43.3 | 42.0 | 4.7 | 4.6 | 43.3 | 42.7 | 33.6 | 30.0 | 53.3 | 48.1 |
| Θ_G_ |  | 30 | 40.0 | 39.5 | 3.9 | 5.1 | 40.6 | 39.4 | 30.4 | 27.5 | 46.3 | 48.8 |
|  |  | 60 | 40.6 | 39.8 | 5.1 | 4.6 | 40.2 | 40.4 | 30.7 | 28.6 | 52.0 | 46.8 |
| Θ_MCC_ |  | 30 | 37.9 | 38.4 | 4.2 | 5.3 | 37.6 | 38.7 | 27.4 | 25.3 | 44.5 | 48.2 |
|  |  | 60 | 38.6 | 38.0 | 5.6 | 4.7 | 37.2 | 38.1 | 27.8 | 27.6 | 51.1 | 45.5 |
| Θ_RWC_ |  | 30 | 34.4 | 35.9 | 4.8 | 5.7 | 33.3 | 36.1 | 23.7 | 20.6 | 42.2 | 46.3 |
|  |  | 60 | 34.5 | 34.8 | 6.9 | 5.4 | 32.6 | 33.9 | 20.8 | 22.7 | 48.3 | 44.3 |
| Θ_PWP_ |  | 30 | 26.0 | 28.3 | 6.7 | 8.4 | 27.0 | 29.9 | 11.1 | 8.2 | 35.8 | 45.3 |
|  |  | 60 | 27.5 | 26.7 | 8.8 | 8.3 | 27.7 | 25.9 | 9.6 | 12.9 | 48.3 | 39.9 |
| ***ρ_d_*** | **g cm^-3^** | 30 | 1.46 | 1.56 | 0.09 | 0.09 | 1.45 | 1.55 | 1.27 | 1.39 | 1.69 | 1.74 |
|  |  | 60 | 1.48 | 1.54 | 0.13 | 0.11 | 1.47 | 1.55 | 1.27 | 1.28 | 1.77 | 1.73 |
| *ρ_s_* |  | 30 | 2.56 | 2.58 | 0.05 | 0.07 | 2.56 | 2.59 | 2.44 | 2.39 | 2.66 | 2.69 |
|  |  | 60 | 2.60 | 2.60 | 0.06 | 0.06 | 2.60 | 2.62 | 2.42 | 2.39 | 2.71 | 2.69 |
| **P** | **% vol.** | 30 | 42.8 | 39.5 | 3.6 | 3.7 | 43.2 | 39.8 | 32.5 | 31.9 | 49.2 | 46.4 |
|  |  | 60 | 43.0 | 41.0 | 4.5 | 4.2 | 43.6 | 41.2 | 34.5 | 30.9 | 51.5 | 50.5 |
| **AWC** | **mm** | 30 | 16.8 | 15.2 | 8.0 | 7.8 | 15.5 | 13.6 | 0.6 | 0.0 | 35.2 | 29.5 |
|  |  | 60 | 13.9 | 16.0 | 7.4 | 9.2 | 11.7 | 15.0 | 0.1 | 1.1 | 32.6 | 38.4 |
| **A_MCC_** | **% vol.** | 30 | 4.97 | 2.79 | 2.30 | 1.15 | 4.83 | 2.30 | 1.78 | 1.33 | 15.89 | 5.42 |
|  |  | 60 | 4.67 | 3.97 | 2.12 | 1.68 | 4.97 | 4.10 | 0.10 | 1.38 | 8.89 | 8.42 |
| **P_G_** |  | 30 | 3.27 | 1.22 | 3.58 | 2.05 | 2.16 | 0.00 | 0.00 | 0.00 | 14.17 | 7.32 |
|  |  | 60 | 3.36 | 2.21 | 3.70 | 2.56 | 2.13 | 1.08 | 0.00 | 0.00 | 10.55 | 8.03 |
| **P_SC_** |  | 30 | 5.61 | 3.59 | 2.41 | 1.51 | 5.17 | 3.42 | 2.86 | 0.00 | 17.26 | 7.43 |
|  |  | 60 | 6.12 | 5.30 | 2.72 | 2.15 | 5.73 | 4.98 | 2.35 | 2.05 | 11.40 | 11.32 |
| **P_C_ : P_G_** | **-** | 30 | 5.40 | 10.26 | 3.29 | 5.70 | 4.69 | 8.72 | 1.14 | 1.41 | 13.87 | 25.78 |
|  |  | 60 | 6.35 | 6.98 | 5.22 | 5.27 | 5.21 | 5.10 | 1.13 | 1.45 | 18.10 | 20.32 |
| **C stock** | **g m^-2^ 10cm^-1^** | 30 | 4678 | 4922 | 940 | 1356 | 4731 | 4569 | 2710 | 2963 | 6111 | 8569 |
|  |  | 60 | 4020 | 3408 | 1019 | 1033 | 3949 | 3185 | 1297 | 1141 | 6334 | 6568 |
