# Supplementary material for: Wooded biocorridors substantially improve soil properties in low-altitude rural benchlands
Source: Heliyon. 2024 Jan 17;10(2):e24381. doi: 10.1016/j.heliyon.2024.e24381 (PMC10835163; doi:10.1016/j.heliyon.2024.e24381)
Supplement: Multimedia component 2 [file mmc2.docx]

Supplementary material 2: Chemical soil properties across research plots in depth of 0-30 differently for the biocorridor (BC) and farm land (FL). For abbreviations of soil parameters see Methods section.

| **parameter** | **units** | **mean** | | **sd** | | **median** | | **min** | | **max** | |
| --- | --- | --- | --- | --- | --- | --- | --- | --- | --- | --- | --- |
|  |  | **BC** | **FL** | **BC** | **FL** | **BC** | **FL** | **BC** | **FL** | **BC** | **FL** |
| **^2^pH/H_2_O** | **-** | 6.57 | 6.71 | 0.74 | 0.56 | 6.80 | 6.80 | 5.30 | 5.50 | 7.60 | 7.80 |
| **^2^pH/KCl** | **-** | 5.91 | 6.00 | 0.87 | 0.69 | 6.20 | 6.00 | 4.40 | 4.60 | 7.10 | 7.20 |
| **^2^P** | **mg kg^-1^** | 22.1 | 28.9 | 22.6 | 21.3 | 11.5 | 22.0 | 5.0 | 5.0 | 88.0 | 82.0 |
| **^2^Mg** | **mg kg^-1^** | 309.3 | 271.1 | 76.8 | 76.5 | 320.5 | 267.5 | 137.0 | 105.0 | 439.0 | 532.0 |
| **^2^Ca** | **mg kg^-1^** | 4504.1 | 4074.0 | 2989.3 | 3036.2 | 3667.0 | 3104.5 | 906.0 | 578.0 | 13460.0 | 12970.0 |
| **^2^K** | **mg kg^-1^** | 227.9 | 237.4 | 172.3 | 139.9 | 176.0 | 204.5 | 87.0 | 84.0 | 879.0 | 741.0 |
| **^2^*CEC*** | **mmol chemeq kg^-1^** | 280.3 | 252.1 | 148.4 | 152.3 | 236.4 | 211.9 | 86.1 | 49.5 | 712.8 | 684.7 |
| **^2^*BS*** | **%** | 88.3 | 90.3 | 8.8 | 5.8 | 92.3 | 91.0 | 66.8 | 73.4 | 98.9 | 99.1 |
| **^1^TOC** | **%** | 3.22 | 3.16 | 0.71 | 0.87 | 3.20 | 2.92 | 1.78 | 1.81 | 4.81 | 5.59 |
| **^2^TOC** | **%** | 2.81 | 2.32 | 1.22 | 0.96 | 2.62 | 2.02 | 1.11 | 0.79 | 5.89 | 4.16 |
| **^2^Nt** | **%** | 0.23 | 0.20 | 0.05 | 0.06 | 0.24 | 0.19 | 0.12 | 0.09 | 0.32 | 0.36 |
| **^2^C/N** | **-** | 11.8 | 11.5 | 3.4 | 4.2 | 10.8 | 10.2 | 7.4 | 7.6 | 24.5 | 30.1 |

^1^ parameter of undisturbed soil sample assessment.

^2^ parameter of mixed soil sample assessment.
