# Supplementary material for: Wooded biocorridors substantially improve soil properties in low-altitude rural benchlands
Source: Heliyon. 2024 Jan 17;10(2):e24381. doi: 10.1016/j.heliyon.2024.e24381 (PMC10835163; doi:10.1016/j.heliyon.2024.e24381)
Supplement: Multimedia component 3 [file mmc3.docx]

Supplementary material 3: Biological soil properties across research plots differently for the biocorridor (BC) and farm land (FL). For abbreviations of soil parameters see Methods section.

| **parameter** | **units** | **mean** | | **sd** | | **median** | | **min** | | **max** | |
| --- | --- | --- | --- | --- | --- | --- | --- | --- | --- | --- | --- |
|  |  | **BC** | **FL** | **BC** | **FL** | **BC** | **FL** | **BC** | **FL** | **BC** | **FL** |
| **N-NH_4_^+^** | **mg N-NH_4_^+^ 100g^-1^** | 0.59 | 0.53 | 0.19 | 0.11 | 0.58 | 0.57 | 0.35 | 0.29 | 1.44 | 0.81 |
| **N-NO_3_^-^** | **mg N-NO_3_^-^ 100g^-1^** | 0.12 | 0.16 | 0.06 | 0.10 | 0.10 | 0.13 | 0.03 | 0.02 | 0.24 | 0.47 |
| **RB** | **mg CO_2_ 100g^-1^ h^-1^** | 0.39 | 0.50 | 0.33 | 0.35 | 0.27 | 0.37 | 0.00 | 0.02 | 1.27 | 1.31 |
| **RG** |  | 1.50 | 2.03 | 0.54 | 0.44 | 1.32 | 2.00 | 0.73 | 1.09 | 2.76 | 2.90 |
| **RN** |  | 0.73 | 0.68 | 0.54 | 0.50 | 0.43 | 0.44 | 0.09 | 0.17 | 1.80 | 1.73 |
| **RNG** |  | 1.77 | 2.33 | 0.81 | 0.60 | 1.60 | 2.17 | 0.76 | 1.54 | 3.46 | 3.77 |
| **RN / RB** | **-** | 6.85 | 2.03 | 24.03 | 3.05 | 1.86 | 1.31 | 0.33 | 0.59 | 163.33 | 19.33 |
| **RG / RB** | **-** | 24.74 | 9.17 | 107.53 | 18.82 | 4.78 | 5.35 | 1.73 | 1.51 | 766.67 | 131.33 |
| **RG / RN** | **-** | 2.89 | 4.49 | 1.88 | 2.53 | 2.68 | 4.45 | 1.35 | 1.10 | 12.94 | 11.94 |
| **RNG / RB** | **-** | 29.02 | 10.92 | 123.39 | 22.44 | 4.97 | 5.17 | 1.57 | 1.88 | 863.33 | 142.67 |
| **RNGRG / RNB** | **-** | 0.67 | 0.96 | 0.46 | 0.54 | 0.61 | 0.91 | 0.01 | 0.06 | 3.07 | 2.91 |
