# Supplementary material for: Wooded biocorridors substantially improve soil properties in low-altitude rural benchlands
Source: Heliyon. 2024 Jan 17;10(2):e24381. doi: 10.1016/j.heliyon.2024.e24381 (PMC10835163; doi:10.1016/j.heliyon.2024.e24381)
Supplement: Multimedia component 4 [file mmc4.docx]

Supplementary material 4: Average values of soil properties at research plots differently for the biocorridor (BC) and farm land (FL). For abbreviations of soil parameters see Methods section.

| **parameter** | **units** | **Vracov** | | **Křižanovice** | | **Radějov** | | **Kuželov** | | **Čertův Mlýn** | | **Hrubá Vrbka** | |
| --- | --- | --- | --- | --- | --- | --- | --- | --- | --- | --- | --- | --- | --- |
|  |  | **BC** | **FL** | **BC** | **FL** | **BC** | **FL** | **BC** | **FL** | **BC** | **FL** | **BC** | **FL** |
| **^1^*ρd*** | **g cm^-3^** | 1.44 | 1.66 | 1.43 | 1.49 | 1.48 | 1.58 | 1.54 | 1.49 | 1.40 | 1.55 | 1.49 | 1.59 |
| **^2^pH/H_2_O** | **-** | 5.91 | 6.77 | 6.64 | 6.77 | 7.18 | 6.81 | 6.49 | 6.38 | 5.86 | 6.03 | 7.32 | 7.51 |
| **^2^pH/KCl** |  | 5.07 | 6.07 | 6.01 | 5.96 | 6.59 | 6.12 | 5.83 | 5.67 | 5.16 | 5.19 | 6.83 | 6.98 |
| **^2^P** | **mg kg^-1^** | 53.4 | 61.3 | 22.7 | 23.6 | 32.2 | 39.7 | 6.8 | 14.0 | 6.8 | 14.1 | 10.7 | 20.8 |
| **^2^Mg** |  | 192.6 | 178.3 | 346.1 | 270.3 | 293.3 | 266.9 | 306.3 | 302.7 | 345.0 | 342.9 | 372.4 | 265.3 |
| **^2^Ca** |  | 1362.6 | 1227.6 | 3542.1 | 3315.9 | 3823.7 | 2710.4 | 6580.7 | 3825.4 | 3266.4 | 3151.3 | 8448.9 | 10213.1 |
| **^2^K** |  | 98.7 | 223.1 | 119.6 | 110.7 | 491.8 | 375.7 | 183.0 | 213.6 | 191.0 | 214.2 | 283.1 | 287.3 |
| **^2^*CEC*** | **mmol chemeq kg^-1^** | 106.5 | 87.1 | 229.5 | 209.6 | 241.1 | 183.5 | 388.9 | 253.9 | 239.0 | 227.4 | 477.1 | 550.8 |
| **^2^*BS*** | **%** | 80.0 | 92.6 | 90.6 | 90.9 | 94.1 | 90.7 | 87.5 | 86.3 | 81.8 | 83.4 | 96.2 | 97.6 |
| **^1^TOC** |  | 2.31 | 2.48 | 3.42 | 2.67 | 3.67 | 4.13 | 2.61 | 3.47 | 3.82 | 3.36 | 3.46 | 2.86 |
| **^2^TOC** |  | 1.29 | 1.02 | 2.43 | 1.88 | 2.29 | 1.99 | 2.72 | 2.95 | 3.00 | 2.23 | 5.15 | 3.84 |
| **^2^Nt** |  | 0.14 | 0.12 | 0.22 | 0.17 | 0.22 | 0.21 | 0.24 | 0.29 | 0.28 | 0.22 | 0.28 | 0.21 |
| **^2^C/N** | **-** | 9.0 | 8.8 | 11.1 | 10.8 | 10.3 | 9.7 | 11.2 | 10.3 | 10.7 | 10.2 | 18.4 | 18.0 |

^1^ parameter of undisturbed soil sample assessment.

^2^ parameter of mixed soil sample assessment.
