# Supplementary material for: Wooded biocorridors substantially improve soil properties in low-altitude rural benchlands
Source: Heliyon. 2024 Jan 17;10(2):e24381. doi: 10.1016/j.heliyon.2024.e24381 (PMC10835163; doi:10.1016/j.heliyon.2024.e24381)
Supplement: Multimedia component 5 [file mmc5.docx]

Supplementary material 5: Average values of biological soil properties at research plots differently for the biocorridor (BC) and farm land (FL). For abbreviations of soil parameters see Methods section.

| **parameter** | **units** | **Vracov** | | **Křižanovice** | | **Radějov** | | **Kuželov** | | **Čertův Mlýn** | | **Hrubá Vrbka** | |
| --- | --- | --- | --- | --- | --- | --- | --- | --- | --- | --- | --- | --- | --- |
|  |  | **BC** | **FL** | **BC** | **FL** | **BC** | **FL** | **BC** | **FL** | **BC** | **FL** | **BC** | **FL** |
| **N-NH_4_^+^** | **mg N-NH_4_^+^ 100g^-1^** | 0.45 | 0.37 | 0.77 | 0.46 | 0.54 | 0.55 | 0.62 | 0.64 | 0.57 | 0.59 | 0.62 | 0.59 |
| **N-NO_3_^-^** | **mg N-NO_3_^-^ 100g^-1^** | 0.10 | 0.14 | 0.09 | 0.11 | 0.10 | 0.16 | 0.20 | 0.31 | 0.07 | 0.09 | 0.18 | 0.14 |
| **RB** | **mg CO_2_ 100g^-1^ h^-1^** | 0.31 | 0.43 | 0.20 | 0.23 | 0.33 | 0.29 | 0.46 | 0.53 | 0.17 | 0.36 | 0.89 | 1.14 |
| **RG** |  | 1.10 | 1.87 | 1.10 | 1.98 | 1.61 | 2.05 | 1.55 | 2.14 | 1.18 | 1.68 | 2.44 | 2.47 |
| **RN** |  | 0.38 | 0.55 | 0.53 | 0.36 | 0.72 | 0.44 | 0.81 | 0.82 | 0.24 | 0.33 | 1.67 | 1.57 |
| **RNG** |  | 1.17 | 1.74 | 1.28 | 2.30 | 2.25 | 2.70 | 1.75 | 2.27 | 1.15 | 1.85 | 3.04 | 3.13 |
| **RN / RB** | **-** | 1.26 | 1.26 | 2.65 | 1.56 | 2.17 | 1.50 | 1.78 | 1.56 | 1.45 | 0.92 | 1.88 | 1.37 |
| **RG / RB** |  | 3.59 | 4.33 | 5.51 | 8.47 | 4.83 | 7.02 | 3.40 | 4.07 | 7.10 | 4.68 | 2.74 | 2.16 |
| **RG / RN** |  | 2.86 | 3.43 | 2.08 | 5.42 | 2.23 | 4.68 | 1.91 | 2.62 | 4.90 | 5.10 | 1.46 | 1.57 |
| **RNG / RB** |  | 3.82 | 4.04 | 6.38 | 9.87 | 6.77 | 9.25 | 3.84 | 4.31 | 6.90 | 5.15 | 3.42 | 2.74 |
| **RNGRG / RNB** |  | 0.85 | 0.74 | 0.44 | 0.75 | 0.65 | 0.88 | 0.63 | 0.68 | 0.67 | 1.20 | 0.66 | 0.92 |
